# Endothelium/Nitric Oxide Mediates the Vasorelaxant and Antihypertensive Effects of the Aqueous Extract from the Stem Bark of *Mammea africana* Sabine (Guttiferae)

**DOI:** 10.1155/2012/961741

**Published:** 2012-09-12

**Authors:** Elvine Pami Nguelefack-Mbuyo, Alain Bertrand Dongmo, Télesphore Benoît Nguelefack, Albert Kamanyi, Pierre Kamtchouing, Théophile Dimo

**Affiliations:** ^1^Laboratory of Animal Physiology and Phytopharmacology, Department of Animal Biology, University of Dschang, P.O. Box 67, Dschang, Cameroon; ^2^Laboratory of Animal Physiology, Department of Animal Biology and Physiology, University of Douala, P.O. Box 24157, Douala, Cameroon; ^3^Laboratory of Animal Physiology, Department of Animal Biology and Physiology, University of Yaounde I, P.O. Box 812, Yaounde, Cameroon

## Abstract

This study evaluates the vasorelaxant and antihypertensive effects of the aqueous extract from the stem bark of *M. africana* (AEMA). AEMA was tested *in vitro* on intact or endothelium-denuded rats' aorta rings precontracted with KCl or norepinephrine in absence or in presence of L-NAME or glibenclamide. The effect of a single concentration (300 **μ**g/mL) of AEMA was also examined on the concentration-response curve of KCl. *In vivo*, the antihypertensive effects of AEMA (200 mg/kg/day) were evaluated in male Wistar rats treated with L-NAME (40 mg/kg/day) for 4 weeks. AEMA relaxed aorta rings precontracted with NE or KCl with respective EC50 values of 0.36 **μ**g/mL and 197.60 **μ**g/mL. The destruction of endothelium or pretreatment of aorta rings with L-NAME shifted the EC50 of AEMA from 0.36 **μ**g/mL to 40.65 **μ**g/mL and 20.20 **μ**g/mL, respectively. The vasorelaxant activity of *M. africana* was significantly inhibited in presence of glibenclamide. AEMA also significantly inhibited the concentration-response curve of KCl. Administered orally, AEMA induced acute and chronic antihypertensive effects and normalized renal NO level. These results show that the vasorelaxant activity of AEMA might be mediated by the activation of the NO-cGMP-ATP-dependent potassium channels pathway and might predominantly account for its antihypertensive effect.

## 1. Introduction

Endothelial dysfunction is the hallmark of essential hypertension and develops as a result of an imbalance between contracting and relaxing factors due to decreased nitric oxide (NO) bioavailability or to oxidized NO. NO plays a pivotal role in the cardiovascular homeostasis by regulating blood pressure through the modulation of vascular basal tone. It also exerts a direct effect on renal tubular function through the inhibition of both Na^+^-K^+^-ATPase and Na^+^/H^+^ exchanger [1, 2]. It is obviously known that the inhibition of NO production by L-arginine analogs like N^*ω*^-L-arginine methyl ester (L-NAME) results in sustained hypertension with renal function impairment [3] that worsens the situation. As this animal model strongly mimics the human essential arterial hypertension, it can be used for the evaluation of new antihypertensive substances. 

*Mammea africana* Sabine is a tree that grows in Africa's tropical rain forests. Concoctions from its stem bark are widely used in African traditional medicine to alleviate various diseases and ailments, including arterial hypertension [4, 5]. Previous investigations showed that *M. africana* is largely constituted of xanthones, coumarins, and flavonoids [6, 7]. Dongmo and coworkers [8] demonstrated the vasodilatory effects of the methylene chloride-methanol extract from the stem bark of *M. africana*. This same extract possesses antioxidant effects and is able to prevent the development of arterial hypertension [9, 10]. It was recently shown that the aqueous extract from the stem bark of the same plant exhibits antioxidant activity and enhances the *in vitro* production of nitric oxide [10]. It was therefore hypothesized that this aqueous extract, which is the form mostly used by traditional healers and the local population, could exhibit vasorelaxant and antihypertensive activities. Hence, the aim of the present study was to evaluate the vasodilatory and the curative antihypertensive effects of the aqueous extract from *M. africana* stem bark in N^*ω*^-nitro-L-arginine methyl ester (L-NAME) hypertensive rats.

## 2. Materials and Methods

### 2.1. Plant Preparation

*M. africana* stem bark was harvested in August 2006 at Lomié (East Region of Cameroon) and identified in the National Herbarium of Cameroon where a voucher's specimen number 4221/SRF/CAM was deposited. The aqueous extract of *Mammea africana* (AEMA) was prepared as described earlier [10]. 

### 2.2. Animals

 Male albino Wistar rats aged 12 to 14 weeks and weighing 180 to 250 g were used in this study. They were bred in colony cages under standard laboratory conditions (12 : 12 h dark/light conditions) with free access to commercial diet and water. All animals' procedures were in accordance with the ethical rules of animals care as described by the law 86/609/CEE of the European Committee Guidelines.

### 2.3. Preparation of Aorta Rings and Experimental Protocols

Animals were sacrificed by cervical dislocation and exsanguinated. The thoracic aorta was rapidly removed and placed in a physiological Krebs solution of the following composition (mM): NaCl 119, KCl 4.7, CaCl_2_ 1.6, MgSO_4_ 1.2, KH_2_PO_4_ 1.2, NaHCO_3_ 25, glucose 11.1, and pH 7.4. The vessel was cleaned of fats and connective tissue and cut into rings of about 3 mm long. For endothelium-denuded aorta, a rough catheter was loosely rubbed in the lumen to destroy the endothelium. Rings were suspended between two stainless steel wires inserted into the lumen in an organ bath chamber containing 10 mL of a Krebs solution maintained at 37°C and continuously bubbled with 95% O_2_ and 5% CO_2_. Aorta rings were stretched with a passive tension of 1 g, and the mechanical activity of the tissue was recorded isometrically with a force displacement transducer (Ugo Basile) connected to a recorder (Unirecord 7050).

After a 60 min equilibration period during which the organ was washed every 15 min, the functional integrity of the endothelium was verified as described by Nguelefack et al. [11]. After this verification, the aorta ring was washed until it returns to its initial tension and then subjected to the following protocols.

 The effects of cumulative concentrations (0.1–700 *μ*g/mL) of AEMA were evaluated on intact aorta or on endothelium-denuded aorta precontracted by KCl (60 mM) or NE (1 *μ*M). The effect of each extract's concentration was observed for 15 min, time within which the effect has reached a plateau, before adding the next concentration. In order to verify if the effects of AEMA on vascular smooth muscle were mediated by NO, cumulative concentrations of AEMA were examined on rings with functional endothelium preincubated for 15 min in 100  *μ*M of L-NAME, an NO synthesis inhibitor. In another set of experiments, protocols were designed to investigate the role of ATP-activated potassium channels (K_ATP_^+^ channels) in the vascular response induced by the AEMA. The aorta rings were preincubated with L-NAME (100 *μ*M) alone or with L-NAME (100 *μ*M) + glibenclamide (10 *μ*M), a K_ATP_^+^ channels blocker for 15 min. The organ was then contracted with 1 *μ*M norepinephrine and relaxed with 300 *μ*g/mL of EAMA. This concentration was able to induce similar relaxant effect in NE- or KCl-induced contraction as well as in presence and in absence of functional endothelium. The implication of voltage-dependent Ca^2+^ channels inhibition in the relaxation induced by AEMA was also evaluated. For this purpose, a cumulative concentration-response curve for KCl (3–120 mM) was constructed in presence and in absence of a single concentration (300 *μ*g/mL) of the plant extract.

### 2.4. *In Vivo* Antihypertensive Test

Animals were acclimatized to the blood pressure measuring equipment for two consecutive days. Then, systolic blood pressure was measured twice, and the average value was used as baseline. They were randomly assigned to four groups of five rats each in such a way that the average baseline blood pressure was approximately the same in different groups. The first group served as control and orally received distilled water for 4 consecutive weeks. The other 3 groups received L-NAME at the dose of 40 mg/kg/day for 2 weeks. At the end of these 2 weeks, treatments continued as follows: group 2 kept on receiving L-NAME for the next 2 weeks, group 3 was treated concomitantly with L-NAME (40 mg/kg/d) and captopril (20 mg/kg/d), while group 4 received L-NAME (40 mg/kg/d) plus AEMA (200 mg/kg/d). At the beginning of this second phase of the treatment, blood pressure was followed for 24 hours after the first administration of captopril or *M. africana*. 

Baseline, acute, and chronic systolic blood pressure (SBP) recordings were made by indirect tail-cuff plethysmography using a Panlab 40254 amplifier coupled with a Tarkan 5213031 recorder. 

### 2.5. Assessment of Renal Function

 Animals were put into individual metabolic cages and acclimatized for two consecutive days prior to captopril or plant extract administration. Urine was collected at week 2 from the beginning of the experiment (baseline) and every week after the commencement of captopril or *M. africana* treatment and stored at −20°C for creatinine, Na^+^, and K^+^ quantification.

 At the end of the experiment, animals were anesthetized by intraperitoneal injection of 1.5 g/kg ethyl carbamate. The abdominal artery was cannulated and blood samples were collected in EDTA-containing tubes and centrifuged at 3500 rpm for 15 min. Aliquots of plasma were frozen at −20°C for further creatinine measurement. Immediately after blood collection, kidneys were removed and used for NO determination. 

 Na^+^ and K^+^ concentrations were measured by flame photometry (JENWAY PFP7). Creatinine was measured using a commercial kit (Chronolab), and absorbencies were red at 500 nm with a Helios epsilon spectrophotometer. The creatinine clearance was calculated and used as an estimate of glomerular filtration rate (GFR) using the following formula:

(1) $$\begin{array}{c} \text{GFR}=\frac{U\times V}{P\times 1440}, \end{array}$$

where *U* is the urine creatinine concentration, *V* is the 24 h urine volume, *P* is the plasma creatinine concentration, and 1440 is the number of minutes in a day [12].

 NO was determined by Griess reaction (1% sulfanilamide and 0.1% naphtylethylene diamine in 2.5% orthophosphoric acid) in renal tissue homogenates.

### 2.6. Drugs

Captopril was purchased from Denk Pharma; glibenclamide, carbachol, KCl, L-NAME, naphtylethylene diamine, norepinephrine, orthophosphoric, acid and sulfanilamide were purchased from Sigma-Aldrich Chemie Gmbh (Taufkirchen, Germany). EDTA and ethyl carbamate were obtained from Fluka (Germany).

### 2.7. Statistical Analysis

Relaxation responses are expressed as the percentage reversal of the force generated by the contractile agent. EC_50_ was determined after logarithmic transformation of the concentration-response curve using GraphPad Prism 4.0 software. Reported values are expressed as means ± SEM. Two-way ANOVA with or without repeated measures followed by Bonferroni posttest was used to assess differences between means for data concerning blood pressure and relaxation, respectively. Data concerning glomerular filtration rate, natriuresis, kaliuresis, body weight, and renal NO content were analyzed using one-way ANOVA followed by Tukey test. 

## 3. Results

### 3.1. Effects of *Mammea africana* on KCl and Norepinephrine (NE) Contractions

**Figure 1:**
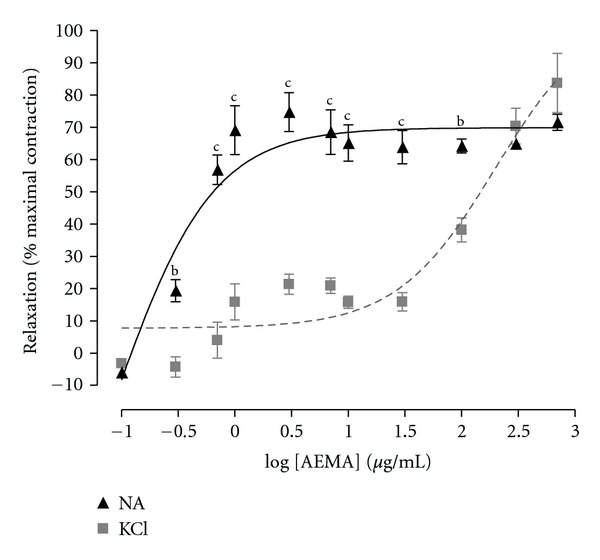
Cumulative concentration-dependent vasorelaxant effects of the aqueous extract of *Mammea africana* (AEMA) on intact aortic rings contracted with KCl (60 mM) or norepinephrine (NE; 1 *μ*M). Each point represents the mean ± SEM, *n* = 6. ^b^ *P* < 0.01; ^c^ *P* < 0.001 compared to the relaxation of the contraction elicited by KCl.

 AEMA induced a concentration-dependent relaxation of aortic rings precontracted with NE (1 *μ*M) or KCl (60 mM) with respective EC_50_ values of 0.36 *μ*g/mL and 197.60 *μ*g/mL. The maximum relaxation effect reached by the plant extract was obtained at the concentration of 3 *μ*g/mL on contraction elicited by NE and at 700 *μ*g/mL on arterial rings contracted with KCl. As depicted in Figure 1, EAMA produced 74.37% relaxation of NE contraction versus 83.71% relaxation of the KCl contraction.

### 3.2. Effect of L-NAME and Endothelium Destruction on *Mammea africana* Vasodilatory Activity

**Figure 2:**
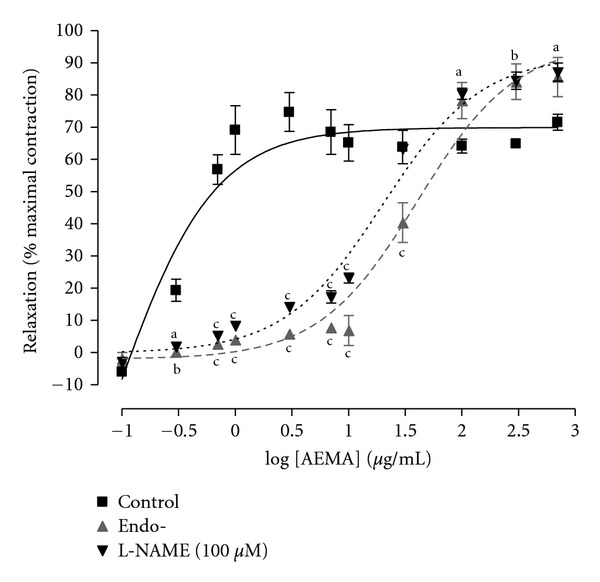
Vasorelaxant effects of the aqueous extract of *Mammea africana* (AEMA) on intact aorta (control), endothelium deprived aorta (endo-), or on intact aorta pretreated with L-NAME (100 *μ*M). Aorta rings were contracted with 1 *μ*M norepinephrine. Data represent the mean ± SEM, *n* = 6. ^b^ *P* < 0.01; ^c^ *P* < 0.001 compared to control.

Endothelium destruction and pretreatment of aortic rings with L-NAME (100 *μ*M), a nitric oxide synthesis inhibitor, significantly inhibited the vasodilatory activity of AEMA on NE-induced contraction as indicated by the increase in EC_50_ value from 0.36 *μ*g/mL in control rings to 40.65 *μ*g/mL and 20.20 *μ*g/mL, respectively. However, neither endothelium destruction, nor L-NAME treatment affected the vasodilatory activity of higher concentrations (≥100 *μ*g/mL) of the plant extract, the plateau being reached at 300 *μ*g/mL (Figure 2). For these reasons, the concentration of 300 *μ*g/mL was used in the following investigations.

### 3.3. Effect of Glibenclamide on* Mammea africana *Vasodilatory Activity

**Figure 3:**
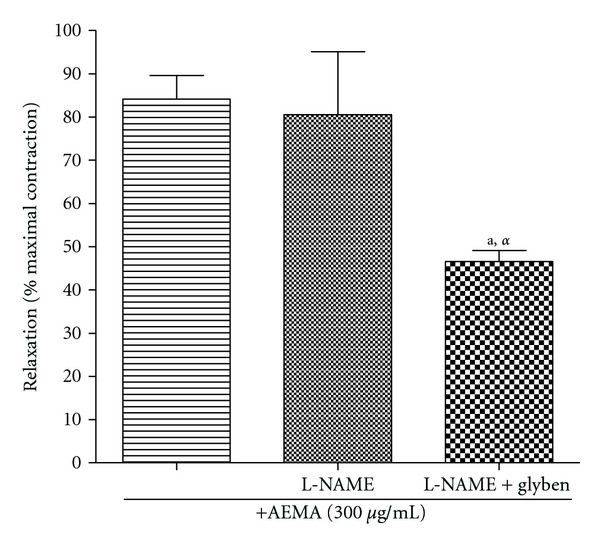
Vasodilatory effect of the aqueous extract of *Mammea africana *(AEMA; 300 *μ*g/mL) in presence of L-NAME (100 *μ*M) alone or in association with glibenclamide (Glyben; 10 *μ*M) on norepinephrine-induced contraction. Data represent the mean ± SEM, *n* = 6; ^a^ *P* < 0.05 compared to the control; ^*α*^ *P* < 0.05 compared to L-NAME.

In presence of L-NAME (100 *μ*M) alone, AEMA (300 *μ*g/mL) induced 80.55% relaxation of the vessel. The addition of glibenclamide (10 *μ*M), an ATP-dependent K^+^ channel blocker in the incubation medium, inhibited the activity of AEMA by 41.87% (Figure 3).

### 3.4. Effect of* Mammea africana *on KCl Contraction

**Figure 4:**
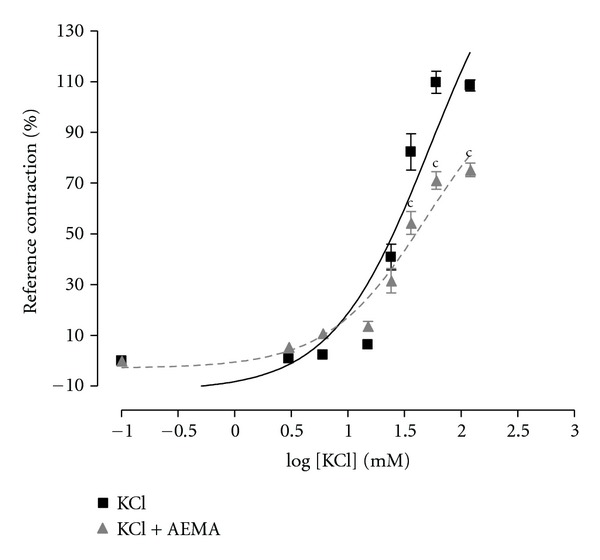
Effect of the aqueous extract of *Mammea africana* (AEMA; 300 *μ*g/mL) on concentration-response curve of KCl. Reference contraction was induced with KCl (60 mM). Data represent the mean ± SEM, *n* = 6; ^c^ *P* < 0.001 compared to KCl.

Cumulative concentrations (3–120 mM) of KCl induced a progressive increase in vascular tension. When the aorta rings were preincubated with the plant extract at the concentration of 300 *μ*g/mL, it caused a reduction in the contractile effect of KCl, reducing the *E*_max⁡⁡_ from 108.47% in control arteries to 75.23% in presence of the AEMA (Figure 4).

### 3.5. Effect of *Mammea africana* on Animal Body Weight and Blood Pressure

**Figure 5:**
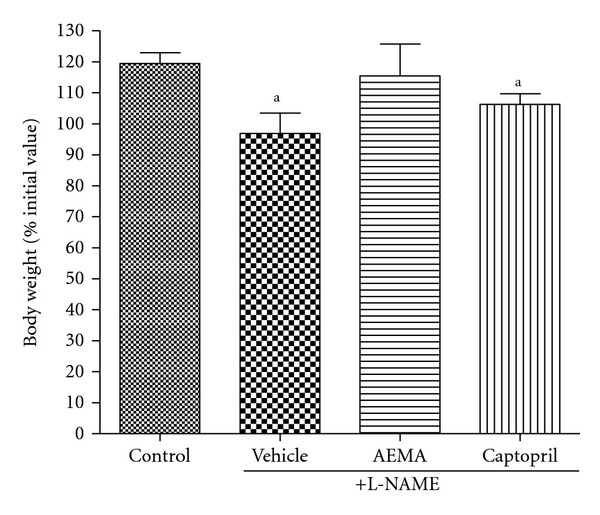
Effect of chronic treatments on the animal relative body weight (% of body weight at the beginning of the experiment). *N* = 5; ^a^ *P* < 0.05, significantly different compared to control.

 As compared to control group, 4 weeks chronic treatment of animals with L-NAME significantly (*P* < 0.05) reduced the normal increase in body weight (96.92% versus 119.53% in control). Animals concomitantly treated with captopril and L-NAME also showed a significant (*P* < 0.05) reduction in body weight. This parameter was instead normalized in animals receiving L-NAME plus AEMA (Figure 5). 

**Figure 6:**
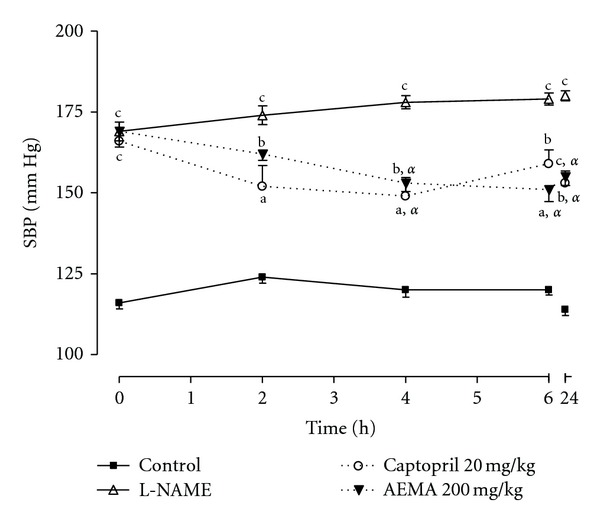
Effect of acute administration of the aqueous extract of *Mammea africana* (200 mg/kg/d) and captopril (20 mg/kg/d) on systolic blood pressure (SBP) of L-NAME (40 mg/kg/d) hypertensive rats. Data represent the mean ± SEM, *n* = 5. ^a^ *P* < 0.05; ^b^ *P* < 0.01; ^c^ *P* < 0.001 compared to control. ^*α*^ *P* < 0.05 compared to L-NAME.

Acute treatment of L-NAME-hypertensive rats with AEMA at the dose of 200 mg/kg induced a time-dependent decrease (*P* < 0.01) in systolic blood pressure (SBP), as compared to untreated hypertensive rats. The progressive fall in SBP started 4 h after oral administration of the plant extract and was maintained throughout the 24 h period. SBP reduced from 169.00 ± 4.84 mmHg before the administration of AEMA to 153.00 ± 2.55 mmHg (9.47%), 4 h after the administration of the plant extract. Captopril (20 mg/kg/d) used as the reference drug induced a similar blood pressure lowering effect (Figure 6).

**Figure 7:**
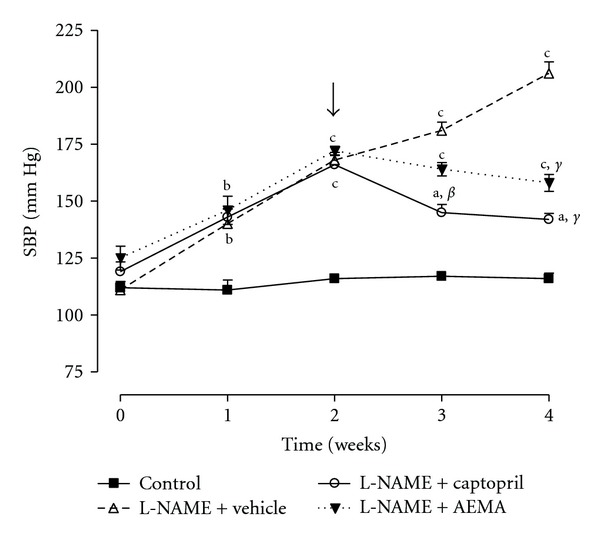
Effect of chronic administration of the aqueous extract of *Mammea africana* (200 mg/kg/d) and captopril (20 mg/kg/d) on systolic blood pressure (SBP) of L-NAME (40 mg/kg/d) hypertensive rats. Arrow indicates the time when the aqueous extract and captopril were introduced. Data represent the mean ± SEM, *n* = 5. ^a^ *P* < 0.05; ^b^ *P* < 0.01; ^c^ *P* < 0.001 compared to control. ^*β*^ *P* < 0.01; ^*γ*^ *P* < 0.001 compared to L-NAME.

Chronic administration of 40 mg/kg/d L-NAME significantly increased (*P* < 0.01) blood pressure from 111.00 ± 3.67 mmHg to 206.00 ± 5.09 mmHg at the end of the four weeks of treatment. The treatment of L-NAME-hypertensive rats with the AEMA for two consecutive weeks resulted in a significant (*P* < 0.001) antihypertensive effect with a 23.30% reduction in hypertension at the end of the experiment compared to untreated L-NAME-hypertensive rats (158.00 ± 3.74 mmHg versus 206.00 ± 5.09 mmHg). Similar to the AEMA, captopril exhibited a potent antihypertensive effect that resulted in 31.07% drop in SBP (142.00 ± 2.55 mmHg versus 206.00 ± 5.09 mmHg) (Figure 7). 

### 3.6. Effects of* Mammea africana *on Renal Function

 There was no significant change in urine output between groups (data not shown). At the end of the second week of treatment, L-NAME induced a transient decrease in kaliuresis, whereas natriuresis remained unchanged as compared to control group. The kaliuresis was completely restored with the prolongation of the L-NAME treatment. Neither AEMA nor captopril significantly affected the natriuresis and the kaliuresis. Glomerular filtration rate (GFR) was not affected by the different treatments although a slight increase in this parameter was observed in L-NAME and AEMA groups. L-NAME administration resulted in a decreased in NO renal level. This reduction was completely reversed by the administration of AEMA in L-NAME hypertensive rats (Table 1). 

## 4. Discussion

Increased peripheral resistance is the common feature found in both human and animal hypertension as a result of decreased NO bioavailability and/or increased secretion of contracting factors. Reducing this peripheral resistance will thus lead to a decrease in blood pressure.

On rat aorta rings precontracted with KCl or norepinephrine (NE), AEMA induced a concentration-dependent relaxation. The vascular smooth muscle contraction elicited by KCl results from increased intracellular calcium concentration through L-type voltage-operated calcium channels activation following plasma membrane depolarization [13], whereas NE stimulates *α*_1_ adrenergic receptors located on vascular smooth muscle to cause Ca^2+^ entry through receptors-operated Ca^2+^ channels [14] and release of Ca^2+^ from internal stores [15]. The vasodilatory activity of the AEMA was more pronounced on NE-induced contraction (EC_50_ = 0.36 *μ*g/mL) compared to that elicited by KCl (EC_50_ = 197.60 *μ*g/mL). These results are in accordance with those obtained by Dongmo and coworkers [8] using the methylene chloride/methanol stem bark extract of *M. africana* and show that the AEMA acts mainly through metabotropic signaling pathway more than through ionotropic pathway. The vascular endothelium plays a pivotal role in the vascular tone regulation thanks to its ability to produce both vasoconstrictor (endothelin-1, angiotensine II, and thromboxane A_2_) and vasodilating (NO, and prostacycline) factors [16, 17].

In order to verify if the vascular endothelium mediates the vasodilatory activity of the AEMA, the effect of this plant extract was examined on endothelium-deprived aorta rings. Results show that the destruction of the vascular endothelium significantly inhibited the relaxation induced by low concentrations of AEMA, suggesting that the effect of the plant extract is at least partially endothelium dependent. 

To determine the endothelial mediator responsible for the endothelium-dependent vasodilatory activity of the AEMA, its effect was tested on intact rings preincubated with L-NAME, an NO synthase inhibitor. The data showed that the pretreatment of aortic rings with L-NAME induced a significant inhibition of AEMA vasodilatory effects, similar to the endothelium destruction. This suggests that the endothelium-dependent vasodilatory activity of the AEMA depends solely upon NO release. It is well known that NO acts on vascular smooth muscle by activating a soluble guanylate cyclase to produce cyclic guanosine monophosphate (cGMP). The cGMP produced further activates PKG which causes myosin light chain phosphorylation [18] or activates the ATP-sensitive potassium channels, both actions leading to smooth muscle relaxation.

 However, it is still obvious that high concentrations of *M. africana* extract exhibit an endothelium-independent activity which suggests a direct effect of the active principles present in the AEMA on vascular smooth muscle. In order to determine the mechanisms concerned with the direct effect of *M. africana* on vascular smooth muscle, the effect of the AEMA was assessed on the concentration-response curve for KCl. At a single concentration of 300 *μ*g/mL, *M. africana* partially inhibits the concentration-response curve for KCl. Two hypotheses emerge from this result: the AEMA could hyperpolarize the plasma membrane, making it difficult to depolarize by a hyperpotasium medium, or it might inhibit the intracellular Ca^2+^ increase.

The first hypothesis was verified by examining the effects of the AEMA (300 *μ*g/mL) in presence of L-NAME alone or in combination with glibenclamide, an ATP-sensitive K^+^ channels blocker on NE-induced contraction. In presence of L-NAME alone, *M. africana* produced 80.55% relaxation of the vessel, whereas in presence of L-NAME and glibenclamide only 46.82% relaxation was obtained. It could be assumed that about 42% of the vasodilatory activity of the AEMA might depend upon ATP-sensitive K^+^ channels activation. Activation of these channels leads to membrane hyperpolarization [19] due to K^+^ extrusion from the cell. Thus, hyperpolarization of the plasma membrane is at least partially responsible for the vasodilatory activity of the AEMA. Since 42% of relaxation induced by the AEMA was inhibited by glibenclamide, this means that the remaining 58% relaxation used another signaling pathway which is most likely the inhibition of the calcium signaling pathway. This alternative is in agreement with the second hypothesis and is supported by the fact that AEMA significantly reduced the amplitude of contraction induced by KCl which mostly depends upon calcium influx through voltage-dependant channels. Taken together, these results suggest that the AEMA causes relaxation of the aortic smooth muscle by a mechanism involving the release of NO by the vascular endothelium, the activation of cGMP-ATP-sensitive K^+^ channels, and probably the reduction of intracellular Ca^2+^ through blockade of calcium influx and/or calcium release from intracellular stores. This vasodilatory activity may account for its antihypertensive effects as observed in acute and chronic treatment in L-NAME hypertensive rats. 

L-NAME-induced hypertension is associated with impaired renal function [3, 20]. It was observed that urine output and urinary Na^+^ and K^+^ excretion in animals treated with L-NAME alone were similar to those of control animals. Diuresis, natriuresis, kaliuresis, and glomerular filtration rate (GFR) were not affected by treatment with the plant extract. This result corroborates our previous findings using the methylene chloride/methanol extract of *M. africana* in a preventive treatment [21] and further shows that the antihypertensive effect of AEMA is not related to any diuretic effect. 

Animals treated concomitantly with L-NAME and AEMA showed completely normalized renal NO, suggesting that AEMA may be able to potentiate the endogenous NO production. In fact, it has been shown that this extract increases the *in vitro* production of NO [10]. The same could be observed *in vivo* and might contribute to the antihypertensive effect of *M. africana*. 

The major finding of this study is that the aqueous extract of the stem bark of *Mammea africana* partially reversed an established hypertension and that vasodilation of arterial smooth muscle may account for its antihypertensive effect. This vasodilatory effect might be mediated by the NO-cGMP-ATP-dependent potassium channels pathway. 

## Figures and Tables

**Table 1:** Effects of chronic treatment (4 weeks) on the natriuresis, kaliuresis, glomerular filtration rate (GFR), and nitric oxide (NO) level in rat kidney.

	Week 2		Week 3		Week 4			
	Na^+^	K^+^	Na^+^	K^+^	Na^+^	K^+^	GFR (mL/kg/min)	NO level (*μ*mol/g)
	(mEq/kg/24 h)	(mEq/kg/24 h)	(mEq/kg/24 h)	(mEq/kg/24 h)	(mEq/kg/24 h)	(mEq/kg/24 h)		
Control	13.60 ± 1.08	14.50 ± 1.03	11.09 ± 0.66	10.86 ± 1.33	12.02 ± 1.22	10.22 ± 1.82	0.10 ± 0.02	1.71 ± 0.19
L-NAME	9.08 ± 1.47	9.94 ± 0.95^a^	11.48 ± 0.76	13.12 ± 0.74	11.55 ± 1.61	12.10 ± 1.62	0.12 ± 0.02	0.96 ± 0.14^a^
L-NAME + Captopril	9.78 ± 0.77	9.17 ± 1.12^a^	11.74 ± 0.93	13.59 ± 1.25	12.84 ± 0.44	14.75 ± 1.22	0.08 ± 0.02	1.45 ± 0.15
L-NAME + AEMA	11.92 ± 0.36	10.48 ± 0.88^a^	12.22 ± 1.18	12.81 ± 1.38	10.61 ± 0.42	11.08 ± 1.06	0.14 ± 0.02	1.70 ± 0.13^*α*^

GFR and NO level were measured at the end of the treatment (4 weeks). Data represent the mean ± SEM, *n* = 5. ^a^ *P* < 0.05 compared to control; ^*α*^ *P* < 0.05 compared to L-NAME.
